# Editorial: Menopause: mood disorders and obesity

**DOI:** 10.3389/fendo.2024.1403692

**Published:** 2024-04-17

**Authors:** Monica Marques Telles, Eliane Beraldi Ribeiro

**Affiliations:** Department of Physiology, Universidade Federal de São Paulo, São Paulo, Brazil

**Keywords:** menopause, ovarian function, obesity, metabolic disorders, mood disorders, osteoporosis, hormone replacement therapy

This Research Topic aimed to provide an overview of the relationship between mood disorders and obesity in the context of menopause. Despite being a physiological process involving oocyte depletion, loss of ovarian steroids, and the permanent cessation of menses, menopause might significantly impact the quality of life of middle-aged and older women. In fact, some of the menopause-related consequences are particularly worrying, such as becoming overweight/obese and its related metabolic diseases, mood disruption, osteopenia/osteoporosis, genitourinary symptoms, vasomotor instability, and insomnia, among others. Given the elevated risks associated with menopausal consequences, coupled with the fact that women are expected to spend at least one-third of their lives in the post-menopausal stage, it is highly necessary to fully understand this physiological phenomenon. This understanding will pave the way for the development of new therapeutic approaches focusing on attenuating menopausal symptoms.

The development of menopause-related mood disorders and obesity has been demonstrated in both animal and human studies. Studies conducted in our laboratory have demonstrated that after eight weeks of ovariectomy, rats exhibited increased energy efficiency, visceral adiposity, body mass, and body mass gain, as well as impaired glucose homeostasis and serotonin-induced hypophagia. These effects were worsened by the intake of a high-fat diet, as expected. Furthermore, ovariectomy also resulted in anxious- and depressive-related behaviors, which were positively associated with hypercholesterolemia and hyperleptinemia, in addition to an impairment in the hypothalamic and hippocampal serotoninergic pathway (1–5). These findings corroborate studies regarding human menopause, which have shown evidence of body weight and adiposity gain, as well as the development of mood disorders such as anxiety and depression (6).

The articles featured in this special edition evidenced important effects of menopause on women´s quality of life. Tang et al. evaluated the associations between obesity or abdominal obesity and menopausal symptoms during the perimenopause period. Although no correlations were found regarding obesity and depression, the authors reported that both conditions were significantly correlated with severe or frequent vasomotor instability (e.g. hot flashes), anxiety, and increased sexual functioning symptoms in Chinese women. Furthermore, this study evidenced that abdominal obesity increases more rapidly than obesity in middle-aged women. In the manuscript by Liu et al., the transcriptome sequencing data combined with the single-cell sequencing data of the ovarian tissue before and after perimenopause was evaluated. It was found that modulation of genes was involved in processes such as the differentiation trajectory of follicular cells in the ovary, granulosa differentiation, and granulosa state maintenance. The authors hypothesized that the state of the granulosa at the end stage was associated with aberrant regulation of estrogen levels. In addition, during menopause progression, a gradual decrease in TGFb and MAPK pathway with a concomitant increase of the p53 pathway activity was discovered, described by the authors as the possible main features of the changes around perimenopause, while vascular inflammation was potentially the major feature of perimenopausal syndrome in the age group of 40-49. Furthermore, the modulation of transcription factors in different stages of life, which could possibly characterize the ovarian status before and after perimenopause, was reported.

Additional consequences of menopause include memory deficits and osteoporosis. In the meta-analysis by Chen et al., it was suggested that menopausal hormone therapy does not improve verbal memory in postmenopausal women and may even impair short-term memory retention. Regarding the link between osteoporosis and menopause, Tang et al. conducted a study using questionnaires from 524 postmenopausal women in Sanya and Hainan provinces in China. The authors reported that, in this population, factors such as low BMI, blood calcium and vitamin D levels, duration of kyphosis, and time spent on outdoor activities are independent risk factors for osteoporosis in postmenopausal women.

Interestingly, the study by Noronha et al., which involved forty-eight postmenopausal women, demonstrated that combined training—integrating aerobic and strength exercises within the same session—significantly improved metabolic indicators of cardiovascular health. This regimen resulted in reduced levels of glucose, triacylglycerol, and cholesterol in circulation. Additionally, a reduction on systolic and diastolic blood pressure and serum concentrations of both mercury and lead were observed. Furthermore, the study reported a modulation of DNA methylation, leading to improved insulin sensitivity and the modulation of gene expression involved in energy metabolism, myogenesis, contractile properties, and oxidative stress.

Concluding this Research Topic, a cross-sectional analysis with 3,526 non-institutionalized American women evidenced that the higher the weight-adjusted-waist index, the higher the probability of infertility (Wen and Li). The authors evaluated data obtained from the National Health and Nutrition Examination Survey (NHANES) 2013-2018 across various demographic situations and the result was stable independently across the region. Given the ease of calculating this index, which involves only two measures–body weight and waist circumference–this study raised the possibility of using the weight-adjusted-waist index as an anthropometric index to predict infertility.

We hope this editorial has contributed to a better understanding of menopause, obesity, and mood disorders. We would like to express our appreciation to all the contributors and reviewers for their help in assembling this engaging and timely collection of articles.

## Author contributions

MT: Conceptualization, Writing – original draft. ER: Conceptualization, Writing – review & editing.
